# Grand Challenges in Materials Science: A Special Issue

**DOI:** 10.3390/ma16186109

**Published:** 2023-09-07

**Authors:** Vuk Uskoković

**Affiliations:** 1 TardigradeNano LLC, 7 Park Vista, Irvine, CA 92604, USA; vuk.uskokovic@tardigradenano.com; 2 College of Engineering, San Diego State University, 5500 Campanile Drive, San Diego, CA 92182, USA

Materials science has and will continue to be a science at a crossroads. It is a type of science that goes hand-in-hand with the engineering of practical solutions but also presents a path toward probing and comprehending the most fundamental phenomena in physics and chemistry. A natural accompaniment of this epistemic and applicative breadth is the constant inflow of challenges and big questions to it. Materials science provides a window to these perennial enigmas but also a view toward glimpsing the solutions.

Sample questions of this grand character that come to mind are as follows:

- What are some of the most exciting topics for research where progress is to be expected in the years and decades to follow?
- Which subjects have been most resistant to progress in the recent past and for what reasons? What strategies may be devised to solve these impasses?
- What explains the demise of certain technologies that were once thought to herald new eras in materials science?
- What are some of the outstanding or inconspicuous problems in materials science, and how could we solve them using theoretical or empirical methods?
- Will a theoretical framework ever be deduced to allow the derivation of synthesis protocols for materials based on their intended functional properties alone?
- How can materials scientists contribute to professional and social egalitarianism and welfare using their influence?
- What are the defining features of creative research in materials science, and how can this creativity be taught?
- What strategies can materials scientists employ to combat the global trend in admiration of research accomplishments to the detriment of depreciation of inventive educational efforts?
- Why is it that materials with extraordinary properties are being produced in the labs and reported in scientific journals like never before in history, yet the quality of materials in daily products is on the decline?
- Which regulatory challenges for advanced materials have emerged in the 21st century, and which ethical issues are associated with them?
- Which interdisciplinary crossings involving materials science should be explored more and how?
- How can materials science benefit from the adoption of models and ideas originating from arts and humanities?
- Sociology and philosophy of science are studied at universities, but what would be sociologic trends and philosophical principles that apply uniquely to materials science?
- How can a more rigorous study of the history of materials science open up the path for its progress in the near and the remote future?

The Special Issue publicized via this editorial note is open to review articles and elaborate essays and commentaries that address these and other big questions in today’s materials science and engineering. The authors should unleash their imagination but stay within the limits of rigor as they communicate their precious insights to the readers. 

